# A compact gas attenuator for the SwissFEL ATHOS beamline realized using additive manufacturing

**DOI:** 10.1107/S1600577523004241

**Published:** 2023-05-30

**Authors:** Claude Pradervand, Colette Rosenberg, Hans-Jörg Eckerlin, Kirsten Schnorr, Andre Al Haddad, Peter Wiegand, Christoph Hess, Nazareno Gaiffi, Luc Patthey

**Affiliations:** a Photon Science Division, Paul Scherrer Institut, Forschungsstrasse 111, Villigen 5232, Switzerland; b Large Research Facilities, Paul Scherrer Institut, Forschungsstrasse 111, Villigen 5232, Switzerland; University of Essex, United Kingdom

**Keywords:** X-ray free-electron lasers, gas attenuator, additive manufacturing

## Abstract

Gas attenuators are important devices providing accurate variation of photon intensity for soft X-ray beamlines. In the SwissFEL ATHOS beamline front-end the space is very limited and an innovative approach has been taken to provide attenuation of three orders of magnitude up to an energy of 1200 eV. Additive manufacturing of a differential pumping system vacuum manifold allowed a triple pumping stage to be realized in a space of less than half a meter. Measurements have shown that the response of the device is as expected from theoretical calculations.

## Abstract 2

A compact gas attenuator for the SwissFEL ATHOS beamline with a custom manifold realized using additive manufacturing is described. First results show that the response is as expected from theoretical calculations.

## 1. Introduction

The SwissFEL soft X-ray free-electron laser (FEL) beamline ATHOS went into user operation in 2021. Its design includes a novel layout of alternating magnetic chicanes and short undulator segments. Together with the APPLE X architecture of undulators, the ATHOS branch can be operated in different modes, producing FEL beams with unique characteristics ranging from attosecond pulse length to high-power modes. The optical transport line distributing the FEL beam to the experimental stations was designed with a whole range of beam parameters in mind. Currently two experimental stations, one for condensed matter and quantum materials research and one for atomic, molecular and optical physics, chemical sciences and ultrafast single-particle imaging, are being installed and going into user operation, respectively (Abela *et al.*, 2019 ▸; Milne *et al.*, 2017 ▸). For the experiments, it is vital to provide a means of varying the intensity of the FEL pulses without the need to retune the machine each time. This is accomplished by introducing a tunable gas attenuator into the beamline.

Gas attenuators and filters are used for several applications in synchrotrons and FELs. One application is to suppress harmonics (Johnson *et al.*, 2009 ▸; Suits *et al.*, 1995 ▸), another as a tunable attenuator for soft X-rays (Ryutov *et al.*, 2008 ▸; Sinn *et al.*, 2012 ▸). Gas attenuators in combination with a differential pumping system used as windowless solutions are compulsory for soft X-rays due to the destructive nature of any solids in the low-energy photon beam. Above ∼1000 eV photon energy, thin solid-state foils can be used in combination with a gas attenuator set to a transmission of around 0.1 in order to protect the thin foils. ATHOS is the low-energy branch of the SwissFEL operating in the photon energy range from 250 eV to 2 keV.

The ATHOS gas attenuator is located in the front-end (accelerator tunnel) around 519 m from the electron gun and 53 m from the last undulator. It allows for transmission of the photons between 1 and below 0.001 at up to 1200 eV. This is achieved by a gas-filled section in the beamline with a defined length and controlled pressure. In order to keep the gas pressure as low as possible (<8 mbar) the gas attenuator will be paired with a solid-state attenuator to attenuate photons with higher energies. As the space is rather limited in the ATHOS front-end, a compact design of the gas attenuator is required.

The ideal gas attenuator would have a box function response, ultra-high vacuum (UHV) on either side and perfect flat and in length defined high-pressure range with a high-absorption-cross-section gas. For an ideal gas attenuator, the Beer–Lambert law would suffice to describe the desired transmission,

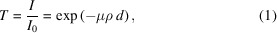

where *I* and *I*_0_ are output and input intensities, respectively, *d* is the length, μ the mass absorption cross section and ρ the density. In order to achieve UHV at either end of the gas attenuators, a differential pumping system (DPS) needs to be implemented. This leads inevitably to a pressure gradient not only in the gas path itself but also over the entire length of the DPS.

## 2. Challenges at SwissFEL – large beam, small space and energy range

In the ATHOS front-end the space is very limited. The overall length of the ATHOS front-end from the last shielding wall after the electron beam dump to the exit port is of the order of 31 m. This is further limited by the main entrance to the SwissFEL accelerator, which needs 5 m of clear path to install/remove undulators or accelerator sections, which therefore leaves a usable length for all components of around 27 m.

Furthermore, due to the low energy range of the ATHOS branch and the various modes of the accelerator and undulators, the beam diameter can be greater than 6 mm (5σ) at 250 eV. The design goal was making the device as compact as possible while reaching a transmission of 10^−3^ up to 1200 eV with gas only, as some experiments require a continual change of the beam intensity.

## 3. Differential pumping system

Differential pumping stages for gas attenuators have been discussed by various groups (Ryutov & Toor, 2000 ▸). In order to achieve a high-pressure drop between the different stages, the conductance should be as low as possible. At higher pressure (above 0.1 mbar), the flow is laminar and roughing pumps must be used. Below ∼0.1 mbar turbomolecular pumps can be used. The formulas below describe the correlation of the various parameters determining the conductance valid for nitro­gen in long tubes.

Pipe conductivity for laminar flow,

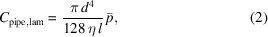

and pipe conductivity for molecular flow,

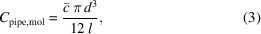

where *C* is the conductivity [l s^−1^], η the dynamic viscosity [Pa s], 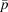 the mean pressure [Pa], *l* the length of the pipe [cm], *d* the diameter of the pipe [cm] and 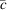 the mean thermal velocity [m s^−1^].

Ideally, the diameter of the pipe should be small, as the conductance is proportional to the third or even fourth power of the diameter. Unfortunately, the constraints already given by the photon beam parameters limit size, *i.e.* the diameter of the pipe must be large enough to let the full beam pass through and the beam size is given by the source. In the case of ATHOS the beam can be greater than 6 mm at low energies. Thus, the smallest apertures have been chosen to be 7 mm to allow for some manufacturing and alignment tolerance. The dynamic viscosity is given by the gas used. The mean pressure can be somewhat effected by using high-performance roughing pumps. The length between the stages should ideally be maximized but is limited by the overall available space.

## 4. Realization

### 4.1. First approach: using Xe

**Figure 1:**
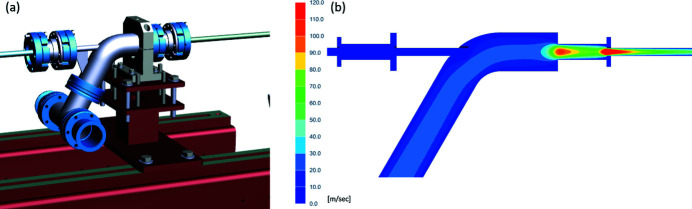
(*a*) Concept of the first differential pumping stage. (*b*) Flow modelling of the differential pumping stage.

As xenon gas has a large mass absorption cross section (as compared with nitro­gen), the first models were designed with a 2 m absorption length using up to 2 mbar gas pressure to keep the design as compact as possible. The gas is injected into the centre of the absorption path. This gives a transmission of 10^−3^ at around 1600 eV at 2 mbar. For the differential pumping system, one roughing pump stage in combination with three turbo pumping stages was foreseen symmetrically on the up- and downstream end of the absorption path. In order to keep the conductivity as small as possible, the lengths of the 7 mm-diameter aperture pipes connecting the pumping stages was 1.3 m to the roughing pump stage, 300 mm to the first turbo pump stage and 250 mm to the second and third stages. Overall, the gas attenuator was around 8.8 m long. To make the first pumping stage as efficient as possible, we designed a special elbow. Modelling the flow showed that a pressure drop of the order of 0.04 could be achieved with the first roughing pump stage (Fig. 1 ▸).

**Figure 2:**
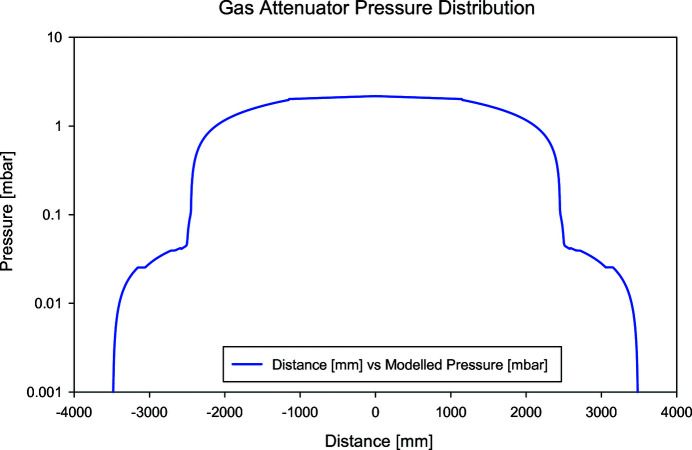
Modelled pressure as a function of distance.

This approach had two major drawbacks. First, the operation cost would have been very high, as the gas usage for continuous operation at 2 mbar was calculated to be around 0.8 l s^−1^. Depending on the usage it was estimated that several gas bottles of xenon per year would be needed for operation. Secondly, due to the long pipe before the first roughing pumping stage, models showed a large, nonlinear gradient for the pressure (Fig. 2 ▸). This was far from the desired box function and would make the calculation of the actual absorption very challenging.

In addition, pumping heavy noble gases such as xenon is particularly critical. The poor heat transfer coefficient of heavy noble gases impedes dissipative cooling and tends to heat up dramatically any mechanical or kinetic transfer pump (Pfeiffer Vacuum, 2013 ▸). Furthermore, even small traces have the potential to contaminate ion pumps further up- and downstream.

### 4.2. Second approach: using nitro­gen gas and additive manufacturing

**Figure 3:**
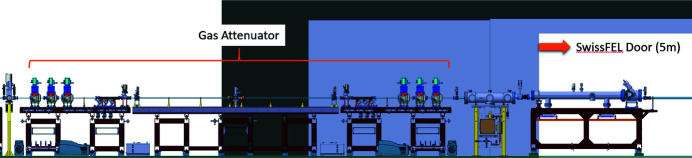
Layout of the gas attenuator in the ATHOS front-end.

Due to the above-mentioned reasons, the operation with xenon was rejected and a new concept was followed. Using nitro­gen gas, it was apparent that the absorption length needed to be increased. Calculations showed that with a 4 m absorption length at least 8 mbar would be necessary to achieve the required transmission of 10^−3^ at 1200 eV. In order to achieve the same vacuum with one pumping stage, the length of the differential pumping stage tube would need to increase by a factor of four at a given pumping power. However, the overall length of the device could not be increased any further, as other components would start to protrude into the access path of the main entrance to the SwissFEL tunnel (Fig. 3 ▸).

**Figure 4:**
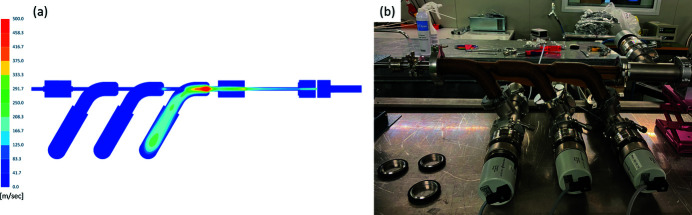
(*a*) Triple differential pumping stage (DPS) simulation. (*b*) Prototype of the triple DPS.

Results from the model above showed that a single stage could not be used to achieve an acceptable pressure drop. Multiple pumping stages would be required, whereas the space was limited to around 0.5 m. Modelling a triple pumping stage showed that the design reduces the pressure enough to add subsequent turbo pumping stages. However, this required a completely different approach to find an acceptable solution for the given space constraints [Fig. 4 ▸(*a*)].

The manifold consists of three chambers, each followed by a 7 mm tube – the first two of length 50 mm and the last one with a length of 62 mm. However, with conventional manufacturing it would be quite challenging and expensive to manufacture custom vacuum chambers with small connecting tubes with the required high accuracy in dimension and concentricity of better than ±0.05 mm. Therefore, we decided to approach the design using additive manufacturing methods, allowing for the most compact solution. A prototype was produced using polyetherketoneketone [Fig. 4 ▸(*b*)]. Initial testing showed promising results reaching 10^−2^ mbar at the exit of the manifold with 9 mbar input pressure to the manifold.

The design was further optimized and printed using stainless steel 1.4404 manufactured by AM Kyburz AG in around 100 h printing time (according to manufacturer). The printed raw part was then stress-free annealed, machined and the CF vacuum flanges were welded to the ports and finally vacuum cleaned.

**Figure 5:**
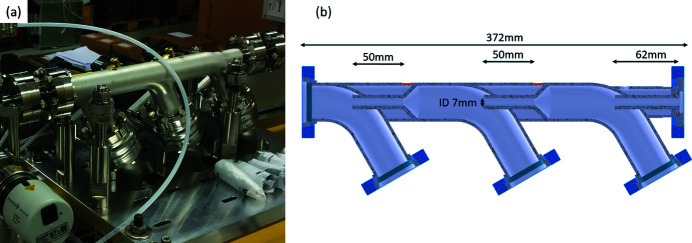
(*a*) 3D printed triple DPS manifold. (*b*) CAD-modelof the triple DPS.

Fig. 5 ▸(*a*) shows the final 3D printed manifold with a total length of only 372 mm. The length of the aperture pipes, with an inner diameter of 7 mm, connecting the gas path to the first roughing pump stages is 96 mm, the pipes between the first and second, and second and third, roughing pump stages are 50 mm, and the exit pipe of the manifold has a length of 62 mm [Fig. 5 ▸(*a*)].

The 3D printed manifold is pumped by three Ebara EV-A06 600 l min^−1^ roughing pumps. Modelling and measurements showed that the pressure after the triple DPS was of the order of 10^−2^ to 10^−3^ mbar, low enough for turbo pumping stages. A 615 mm-long pipe connects to the first turbo pumping stage. This is followed by two more turbo pumping stages connected by 96 mm-long pipes each. All connecting pipes have an inner diameter of 7 mm. The first and second turbo stages consist of two Pfeiffer HiPace300 pumps with the third stage realized with two HiPace80 pumps. Overall, the gas attenuator including the 4 m gas path and all differential pumping stages is 8.8 m long.

## 5. Measurements

**Figure 6:**
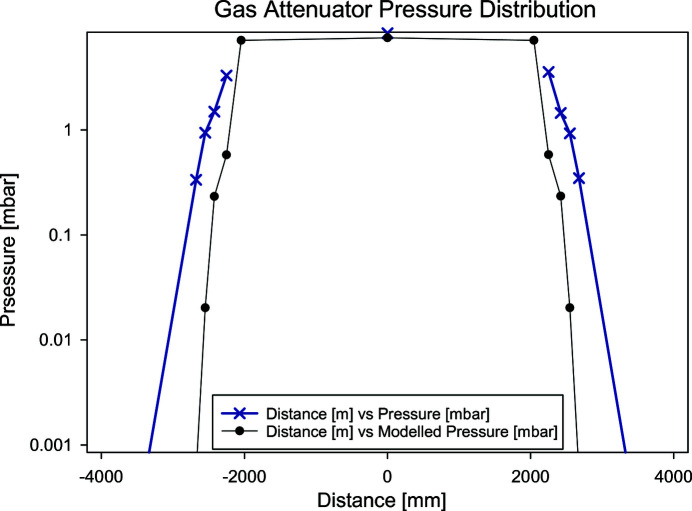
Pressure distribution measured at 8.3 mbar and modelled for 7.5 mbar.

Fig. 6 ▸ shows the pressure distribution over the entire length of the gas attenuator from the model and measurement. As there is no pressure gauge at the points at ±2000 ▸ mm, the measurements are missing. The measurement was performed at an inlet pressure of 8.3 mbar, the model with a pressure of 7.5 mbar.

Within the first half meter at either ends of the gas path, the pressure already drops by one order of magnitude, which corresponds to a transmission change of a factor of 700 at 1 keV. The effects of the nonlinear tails are thus negligible leaving an almost ideal box response in the pressure profile.

Using the Beer–Lambert law, we calculated the transmission for a given pressure and measured the response of the gas attenuator against total photoemission yield for a rare gas using an electron spectrometer (SPECS PHOIBOS 150 EP) at the Maloja endstation. Photoemission yield measurements were chosen due to the linearity of the process over a large dynamic range of incoming photon flux.

**Figure 7:**
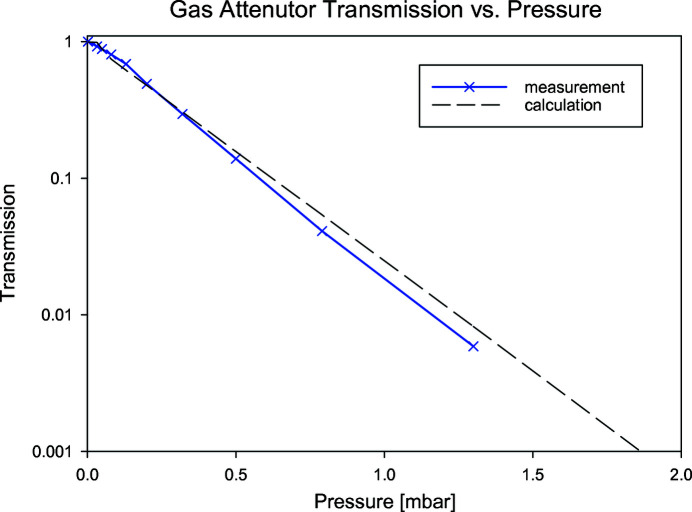
Calculated versus measured transmission.

Fig. 7 ▸ shows the calculated versus measured transmissions. The incoming photon flux was measured with a gas monitor (XGM) in the front-end before the gas attenuator (Tiedtke *et al.*, 2008 ▸). The beam parameters were set to a photon energy of 710 eV (1% bandwidth) and a pulse energy of 980 µJ. The transmitted X-ray intensities were measured with a photoelectron spectrometer using neon gas at around 5 × 10^−6^ mbar measuring Ne 1*s* electrons. Based on this measurement, we confirm that the effective length of the attenuator is 4 m, which supports the idea of a box function response.

A control software considering the Beer–Lambert law was implemented in order to control the gas attenuator by entering the desired transmission directly. The control software considers the 4 m-long attenuator effective length and box function response. The linearity of the attenuator is assessed by measuring the relative X-ray intensity after the attenuator at two different photon energies.

**Figure 8:**
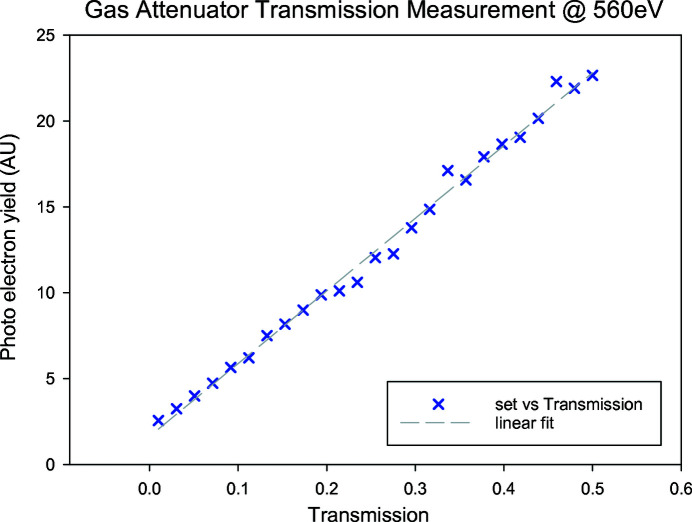
Measurement of the photoelectron yield as a function of transmission for the gas attenuator at 560 eV.

**Figure 9:**
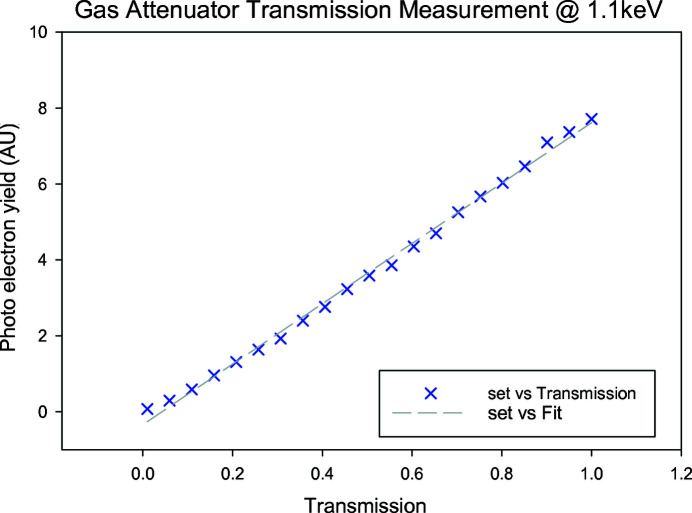
Measurement of the photoelectron yield as a function of transmission for the gas attenuator at 1.1 keV.

Fig. 8 ▸ shows a linearity measurement changing the transmission from 0.01 to 0.5 at a photon energy of 530 eV for the monochromatic beam (0.5 eV bandwidth). Fig. 9 ▸ shows a linearity measurement changing the transmission from 0.05 to 1 at a photon energy of 1.1 keV (1% bandwidth) beam. Again, the incoming photon flux was measured with the XGM and the attenuated photons were measured with the photoelectron spectrometer using neon at around 5 × 10^−6^ mbar measuring Ne 2*s* and 2*p* electrons and integrating 1000 pulses for the 530 eV data and measuring Ne 1*s* electrons and integrating 2000 ▸ pulses for the 1.1 keV data.

## 6. Conclusions

The ATHOS gas attenuator has been commissioned for operation with nitro­gen and is regularly used for experiments at the Maloja and Furka endstations. Thanks to additive manufacturing, the design could be kept as short as possible and fits the space constraints given within the ATHOS front-end. Operation is very stable and changing attenuation can be performed continuously. Even a change from maximum attenuation to full beam will be achieved in around one minute. The attenuator follows the theory very well and allows the attenuator to be set based on a pre-calculated pressure. The use of argon and neon gas is also foreseen in the future and further commissioning is needed. Argon will be used to cover the nitro­gen edge at 407 eV and neon will be used to calibrate the grating monochromator.
